# Female Urgency, Trial of Urodynamics as Routine Evaluation (FUTURE study): a superiority randomised clinical trial to evaluate the effectiveness and cost-effectiveness of invasive urodynamic investigations in management of women with refractory overactive bladder symptoms

**DOI:** 10.1186/s13063-021-05661-3

**Published:** 2021-10-26

**Authors:** M. Abdel-fattah, C. Chapple, K. Guerrero, S. Dixon, N. Cotterill, K. Ward, H. Hashim, A. Monga, K. Brown, M. J. Drake, A. Gammie, A. Mostafa, U. K. Bladder Health, S. Breeman, D. Cooper, G. MacLennan, J. Norrie

**Affiliations:** 1 Aberdeen Centre for Women’s Health Research, Division of Applied Health Sciences, University of Aberdeen, Aberdeen, UK; 2 Department of Urology, Sheffield Teaching Hospitals NHS Foundation Trust, Sheffield, UK; 3 Department of Urogynaecology, NHS Greater Glasgow and Clyde, Glasgow, UK; 4 Health Economics and Decision Science, University of Sheffield, Sheffield, UK; 5 Bristol Urological Institute, North Bristol NHS Trust, Bristol, UK; 6 Faculty of Health and Applied Sciences, University of the West of England, Bristol, UK; 7 Warrell Unit, Manchester University NHS Foundation Trust, Manchester Academic Health Science Centre, Manchester, UK; 8 Bristol Urological Institute, University of Bristol, Bristol, UK; 9 Department of Gynaecology, University Hospital Southampton NHS Foundation Trust, Southampton, UK; 10 Department of Gynaecology, Newcastle Upon Tyne Hospitals NHS Foundation Trust, Newcastle, UK; 11 Bladder Health UK, Registered charity, Birmingham, UK; 12 Centre for Healthcare Randomised Trials, University of Aberdeen, Aberdeen, UK; 13 Edinburgh Clinical Trials Unit, Usher Institute, University of Edinburgh, Edinburgh, UK

**Keywords:** Female, Urodynamics, Overactive bladder, Randomised controlled trial, Uroflowmetry, Filling cystometry

## Abstract

**Background:**

Overactive bladder (OAB) syndrome is a symptom complex affecting 12–14% of the UK adult female population. Symptoms include urinary urgency, with or without urgency incontinence, increased daytime urinary frequency and nocturia. OAB has a negative impact on women’s social, physical, and psychological wellbeing. Initial treatment includes lifestyle modifications, bladder retraining, pelvic floor exercises and pharmacological therapy. However, these measures are unsuccessful in 25–40% of women (refractory OAB). Before considering invasive treatments, such as Botulinum toxin injection or sacral neuromodulation, most guidelines recommend urodynamics to confirm diagnosis of detrusor overactivity (DO). However, urodynamics may fail to show evidence of DO in up to 45% of cases, hence the need to evaluate its effectiveness and cost-effectiveness. FUTURE (Female Urgency, Trial of Urodynamics as Routine Evaluation) aims to test the hypothesis that, in women with refractory OAB, urodynamics and comprehensive clinical assessment is associated with superior patient-reported outcomes following treatment and is more cost-effective, compared to comprehensive clinical assessment only**.**

**Methods:**

FUTURE is a pragmatic, multi-centre, superiority randomised controlled trial. Women aged ≥ 18 years with refractory OAB or urgency predominant mixed urinary incontinence, and who have failed/not tolerated conservative and medical treatment, are considered for trial entry. We aim to recruit 1096 women from approximately 60 secondary/tertiary care hospitals across the UK. All consenting women will complete questionnaires at baseline, 3 months, 6 months and 15 months post-randomisation. The primary outcome is participant-reported success at 15 months post-randomisation measured using the Patient Global Impression of Improvement. The primary economic outcome is incremental cost per quality-adjusted life year gained at 15 months. The secondary outcomes include adverse events, impact on other urinary symptoms and health-related quality of life. Qualitative interviews with participants and clinicians and a health economic evaluation will also be conducted. The statistical analysis of the primary outcome will be by intention-to-treat. Results will be presented as estimates and 95% CIs.

**Discussion:**

The FUTURE study will inform patients, clinicians and policy makers whether routine urodynamics improves treatment outcomes in women with refractory OAB and whether it is cost-effective.

**Trial registration:**

ISRCTN63268739. Registered on 14 September 2017.

## Administrative information

Note: the numbers in curly brackets in this protocol refer to SPIRIT checklist item numbers. The order of the items has been modified to group similar items (see http://www.equator-network.org/reporting-guidelines/spirit-2013-statement-defining-standard-protocol-items-for-clinical-trials/).

**Table:** 

Title {1}	Female Urgency, Trial of Urodynamics as Routine Evaluation (FUTURE study); a superiority randomised clinical trial to evaluate the effectiveness and cost effectiveness of invasive urodynamic investigations in management of women with refractory overactive bladder symptoms.
Trial registration {2a and 2b}.	ISRCTN63268739. Registered on 14^th^ September 2017. Prospectively registered.
Protocol version {3}	Version 10.0; 01/07/2021
Funding {4}	National Institute of Health Research (NIHR), Health Technology Assessment (HTA) programme; Project number 15/150/05
Author details {5a}	^1^Aberdeen Centre for Women’s Health Research, Division of Applied Health Sciences, University of Aberdeen, Aberdeen, UK. ^2^Department of Urology, Sheffield Teaching Hospitals NHS Foundation Trust, Sheffield, UK. ^3^Department of Urogynaecology, NHS Greater Glasgow and Clyde, Glasgow, UK. ^4^Health Economics and Decision Science, University of Sheffield, Sheffield, UK. ^5^Bristol Urological Institute, North Bristol NHS Trust, Bristol, UK. ^6^Faculty of Health and Applied Sciences, University of the West of England, Bristol, UK ^7^Warrell Unit, Manchester University NHS Foundation Trust, Manchester Academic Health Science Centre, Manchester, UK. ^8^Bristol Urological Institute, University of Bristol, Bristol, UK. ^9^Department of Gynaecology, University Hospital Southampton NHS Foundation Trust, Southampton, UK. ^10^Department of Gynaecology, Newcastle Upon Tyne Hospitals NHS Foundation Trust, Newcastle, UK. ^11^Bladder Health UK, Registered charity, Birmingham, UK ^12^Centre for Healthcare Randomised Trials, University of Aberdeen, Aberdeen, UK. ^13^Edinburgh Clinical Trials Unit, Usher institute, University of Edinburgh, Edinburgh, UK.
Name and contact information for the trial sponsor {5b}	Co-sponsor 1. University of Aberdeen, Foresterhill House Annexe, Foresterhill, Aberdeen, AB25 2ZB. researchgovernance@abdn.ac.uk Co-sponsor 2. NHS Grampian Foresterhill House Annexe, Foresterhill, Aberdeen, AB25 2ZB. researchgovernance@abdn.ac.uk
Role of sponsor {5c}	The sponsor played no part in study design; and will play no part in the collection, management, analysis, and interpretation of data; writing of the report; and the decision to submit the report for publication.

## Introduction

### Background and rationale {6a}

Overactive bladder (OAB) syndrome has been defined by the International Continence Society (ICS) as urinary urgency, with or without urgency urinary incontinence, usually with increased daytime frequency and nocturia, in the absence of any other pathology [1].

The Leicestershire MRC (Medical Research Council) Incontinence Study showed a 21% overall prevalence of OAB in women aged ≥ 40 years in the United Kingdom (UK); UUI and mixed urinary incontinence (MUI) represented 11% and 36% of these women respectively [2]. The Epidemiology of Lower Urinary Tract Symptoms (EpiLUTS) study reported relatively higher UUI prevalence rates of 13.3% for men and 30.3% for women in the USA [3]. In 2016, Komeso reported a large epidemiological study showing that the prevalence of urinary incontinence (UI) increases with age; this was most apparent for UUI and MUI: the odds of occurrence of UUI were 2- and 9-fold increased in the 7th and 10th decades, compared with the 6th decade (OR 2.18; 95%CI = 1.5–3.15 and OR = 9.19; 95%CI = 5.56–15.20) respectively [4]. The prevalence of MUI also significantly increased in the 8th to10th decades (both *P* ≤ 0.005), but interestingly, the prevalence of stress urinary incontinence (SUI) did not seem to increase with age in this study. Similar results were shown by the EPINCONT study of 28,000 women [5]. The EPIC prevalence data estimates that the worldwide number of adults aged ≥ 20 years with UUI or MUI was 103 million in 2008, with projected increase to 127 million in 2018 [6]. Therefore, the prevalence of OAB/MUI is likely to increase in the years to come, especially given the ageing population in the UK.

OAB and UUI have been shown to have a negative impact on a woman’s social, physical and psychological wellbeing, leading to embarrassment, low self-esteem and negative effects on the productivity of working women. In extreme cases, women reported avoiding employment because of fear of embarrassing situations [2, 7]; 60% avoided going away from home; and 50% reported avoidance of sexual activity [8]. This debilitating social problem has significant cost implications to the health resources in the UK. The total annual cost to the National Health Service (NHS) is £301 million or 0.3% of the total NHS budget in 2009 [9]. Costs borne by women and their families (e.g. for containment products) were £230 million [10]. Health-related costs for management of OAB and UUI was estimated at approximately €7.0 billion in 2005 across 6 countries: Canada, Germany, Italy, Spain, Sweden and the UK [6].

The National Institute for Health and Care Excellence (NICE) shows the standard benchmark rate for a referral into a UI service for UK women is 0.8% (800 per 100,000 adult female/ year) [11]. In women diagnosed with OAB, the NICE Guideline CG171 recommends initial conservative treatment which includes the following: lifestyle modifications, bladder training and pelvic floor exercise and pharmacological therapy (anticholinergics and/or beta-3 agonist). However, these measures are unsuccessful for approximately 25–40% of women (i.e. refractory OAB) [12]. For these women, NICE recommends “urodynamics” investigation to confirm the diagnosis of detrusor overactivity (DO) before proceeding to invasive treatments such as Botulinum toxin-A injection (BoNT-A) or sacral neuro-modulation (SNM) [11]. NICE CG171 was the relevant guideline at time of the FUTURE study planning; however, the recommendation has not changed in the updated NICE NG123 [13].

Invasive urodynamics is a diagnostic test that involves the insertion of one to two catheters into the bladder and another one into the vagina or the rectum. The rationale for urodynamics is to reproduce the women’s symptoms and to identify the underlying pathology. During bladder filling, DO may be seen; these are uninhibited bladder contractions, which hinder effective urine storage, and are frequently associated with urgency and/or UUI. Urodynamic stress incontinence (USI) may also be seen, and if USI and DO incontinence (DOI) are both present, the woman is diagnosed with MUI. Urodynamics can also identify other pathology, for example bladder outlet obstruction or detrusor underactivity, which may influence the choice of therapy.

Although urodynamics is currently the recommended investigation in the NICE guidelines for the assessment of women with refractory OAB and/or MUI [11, 13], the clinical and cost-effectiveness of urodynamics have not been demonstrated in well designed, adequately powered clinical trials. Interestingly, some of the current evidence on the value of urodynamics in these women suggests little impact, if any, on the post-treatment patient-reported outcomes [14].

### Invasive treatments for refractory OAB

Current guidelines recommend BoNT-A or SNM as the treatments for women with refractory OAB following failure of conservative and medical treatment [11, 13].

BoNT-A treatment is an injection into the bladder wall using a cystoscope (rigid or flexible), either under general or more commonly local anaesthesia. The treatment, if successful, is usually repeated every 6 to 12 months.

In women with refractory OAB and associated DO on urodynamics, Brubaker et al. showed that approximately 60% who received BoNT-A had a positive clinical response on the Patient Global Impression of Improvement scale (PGI-I) [15]. Secondary analyses from two recent randomised controlled trials (RCTs) of BoNT-A versus placebo suggested that successful treatment outcomes did not appear to be related to the pre-operative urodynamics diagnosis of DO [14, 16]. Chapple et al., in a double-blind, placebo-controlled RCT, showed that BoNT-A significantly improves all symptoms of refractory OAB and quality of life (QoL); there was no impact of the pre-operative diagnosis of DO on the treatment outcomes [16]. Similarly, Rovner et al. in a placebo-controlled RCT showed 57% of the patients were satisfied compared to 19% placebo at 3 months following BoNT-A treatment, irrespective of the presence of DO on urodynamics [14]. BoNT-A is now licensed in the UK for treatment of idiopathic refractory OAB symptoms (with symptoms of urinary incontinence, urgency and frequency) without the need for pre-operative urodynamics [17].

In a recent observational study embedded within the BUS RCT, 666 women with non-refractory OAB underwent urodynamics; the results suggested that clinicians and patients appeared to be guided in part by the urodynamics diagnosis in selecting treatment options [18]. Several confounding influences were identified, such as natural fluctuation of disease state, regression to the mean and Hawthorne effects. The economic modelling within the BUS study suggested that urodynamics can be a cost-effective diagnostic strategy for women with predominant symptoms of OAB [18]. However, this was based on fewer women undergoing invasive treatment in the urodynamics group. The authors reported significant cost savings in the urodynamics group associated with a small reduction in clinical effectiveness. It is important to highlight that the BUS study assessed a different cohort of women with significantly milder OAB symptoms and therefore the results could not be generalised to women with refractory OAB.

The principle of SNM is that electrical stimulation of the sacral reflex pathway will inhibit the reflex behaviour of the bladder. SNM is a two-stage procedure; stage one is a SNM test using either a temporary or permanent lead, connected to an external stimulator, while the second stage involves the placement of a subcutaneous implantable pulse generator (permanent implant). If a patient reports at least 50% improvement of the refractory OAB symptoms during the test phase, as recorded in the bladder diaries, they are offered the permanent implant. SNM has the unique advantage that patient outcomes are assessed before a commitment is made to the permanent procedure.

Three RCTs comparing SNM to placebo showed that 52% of patients were dry at 18 months and a further 24% reported at least 50% reduction in leakage episodes (*n* = 58); at 3 years, 46% were dry and 13% improved [19–21]. In one RCT, patients with urgency and frequency showed improvements in several SF-36 domains in the active treatment group (*n* = 51; 90% women) at 6 months follow-up [21]. NICE concluded that following SNM, up to two-thirds of patients achieve continence or substantial improvement in symptoms, with the beneficial effects lasting for up to 3–5 years after implantation [11]. Around one third of patients may require reoperation, most often due to pain at the implant site, infection, or the need for adjustment and modification of the lead system. Interestingly, while urodynamics investigation is considered a standard practice prior to SNM treatment in NICE CG171 and NG123, confirmation of DO is not [11, 13]. One recent observational study reported that pre-operative diagnosis of DO was not a prerequisite selection criterion for SNM [22].

### Sequence of treatment in women with refractory OAB

The best sequence of interventions for women with refractory OAB is not known.

In 2013, NICE CG171 included a heath economic evaluation which suggested that BoNT-A was a cost-effective intervention, in comparison with either no active treatment or SNM and NICE recommended offering BoNT-A as first intervention to women with refractory OAB and DO [11]. They recommended SNM for women unable to catheterise or who have a cultural or ethical objection to catheterisation (slightly amended in NG123 to women unprepared to accept risks of clean intermittent self-catheterisation (CISC) with BoNT-A), or those with persistent symptoms following BoNT-A treatment) [11, 13].

Interestingly, evidence from one recent study highlighted that 61% of women receiving BoNT-A discontinued their treatment at 3 years while 64% discontinued at 5 years [23]. Most recently, Marcelissen et al. showed that only 30% of their patients initiated on BoNT-A treatment were still on treatment at minimum follow-up of 5 years; the majority of patients who discontinued treatment (98%) did so after the 1st or 2nd injection [24]. In an economic model comparing SNM with BoNT-A over a 5-year period with a societal perspective, Leong et al. reported a greater gain in quality-adjusted life years (QALY) and a greater associated cost savings when patients were initiated on SNM treatment [25]. As the QALY gain from BoNT-A injection was lower due to the loss of effect with reinjections over time, SNM became cost-effective after 5 years compared with BoNT-A, with an incremental cost-effectiveness ratio of 27,991 Euros, within the accepted NICE threshold of £20,000 to £30,000.

Accordingly, UK practice varies and usually relies on treatment options available locally within the units. Our brief survey of the potential collaborating centres for the FUTURE study suggests a considerable number of units and surgeons offer BoNT-A treatment for women with refractory OAB with and without urodynamics evidence of DO. In addition, in tertiary units where SNM may be readily available, surgeons tend to offer women with confirmed DO the choice between BoNT-A or SNM after discussion by the local multidisciplinary team (MDT). Some surgeons indicated that they favour SNM in younger patients and/or those with associated voiding dysfunction or faecal incontinence.

In summary, the current evidence highlights the uncertainties and the need for a robust RCT to address this important research question which was prioritised by the NICE guideline CG171 research recommendations: “Further research is needed to answer the question of whether the use of invasive urodynamics, prior to initial or subsequent treatments, affects the outcomes and cost-effectiveness of interventions in women with UI or OAB” [11].

#### Rationale for the trial

NICE recommends urodynamics investigation to confirm the diagnosis of DO in women with refractory OAB before proceeding to invasive treatment [11].

For clinicians, urodynamics is traditionally considered to inform the counselling of women on the chances of success of subsequent treatments. However, in women with refractory OAB, urodynamics fails to show evidence of DO in up to 45% [26]. The accuracy of urodynamics relies on well-calibrated equipment, experience of investigators and their interpretation of a number of subjective parameters. Standardisation of urodynamics is difficult and is influenced by wide variation in staff practice and equipment used [27]. These factors raise a valid debate on the clinical and cost-effectiveness of urodynamics and whether it actually improves the outcomes of subsequent treatments compared to treatment guided by comprehensive clinical assessment only.

From the patients’ perspective, many describe urodynamics as an invasive and embarrassing investigation and associated with an element of emotional distress [28, 29]. Urodynamics is also associated with a risk of discomfort and urinary tract infection [18]. However, the majority of women find it acceptable if it will improve their outcomes post-treatment [18, 30–32]. Unfortunately, the urodynamics test may not replicate the patients’ symptoms in their day to day lives which questions the validity of the treatment options offered based on its results.

For policy makers, inevitably urodynamics is costly to the NHS, including purchase of equipment and disposables, and the need for specialist staff. The urodynamics tariff was £256/patient at the time of the development of the study protocol. Policy makers are faced with the current pressure on health resources in the UK; therefore, there is a pressing need to direct resources towards evidence-based interventions that are proven to positively improve treatment outcomes.

Urodynamics is one such test that has been embedded in clinical practice without robust evidence of its clinical or cost-effectiveness. Current evidence shows urodynamics to have no impact on the patient-reported outcomes following conservative treatment of UI [33] and for those undergoing surgical treatment for symptoms of pure SUI [34]. Accordingly, NICE CG171 has prioritised research to assess the clinical and cost-effectiveness of urodynamics in treatment of refractory OAB [11].

The outcome of the FUTURE study would inform patients, clinicians and policy makers whether routine urodynamics investigation improves the treatment outcomes in women with refractory OAB and whether it is cost-effective.

## Objectives {7}

### Research question

Does routine urodynamics investigation in addition to comprehensive clinical assessment improve patient-reported outcomes following treatment, compared to comprehensive clinical assessment only, in women with refractory OAB symptoms, and is it cost-effective?

### Hypothesis

In women with refractory OAB, urodynamics and comprehensive clinical assessment is associated with superior patient-reported outcomes following treatment and is more cost-effective, compared to comprehensive clinical assessment only.

The primary objectives are to:

1. Evaluate whether routine urodynamics investigation and comprehensive clinical assessment significantly improves patient-reported success rates following treatment, compared to comprehensive clinical assessment only;

2. Assess the cost-effectiveness of routine urodynamics investigation and comprehensive clinical assessment, compared to comprehensive clinical assessment only.

Secondary objectives are to:

1. Assess the above outcomes in pre-specified subgroups of women: OAB and urgency predominant MUI.
2. Explore the clinicians’ attitudes towards urodynamics investigation and its influence on surgical decision-making;
3. Explore the participants attitudes and experience in both pathways;
4. Explore the clinical and cost-effectiveness of the different sequence of treatments for refractory OAB.

## Trial design {8}

**Fig. 1:**
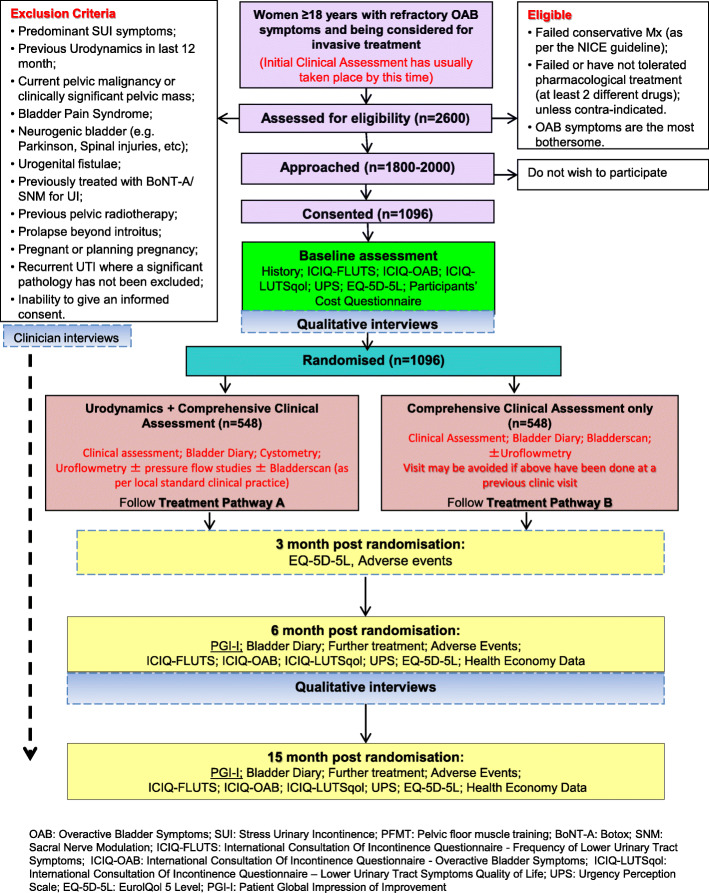
Flow diagram illustrating the participants journey through the FUTURE trial

This is a pragmatic, multi-centre, superiority RCT comparing the effectiveness and cost-effectiveness of routine urodynamics investigation and comprehensive clinical assessment versus comprehensive clinical assessment only in the management of women with refractory OAB symptoms (Fig. 1). An internal pilot study with stop/go criteria is embedded within the RCT to establish whether the projected recruitment rate is achievable.

The trial design includes an economic evaluation from a National Health Service (NHS) perspective, using both a within trial timeframe and a modelled patient lifetime timeframe. Unit costs will be taken from standard sources (NHS Reference Costs, British National Formulary and ‘Unit Costs of Health and Social Care’). Costs and outcomes are discounted at 3.5%. An embedded qualitative component is also included in the trial design to evaluate the patients’ attitudes to, and experiences of, invasive urodynamic testing, and also clinicians’ views on the influence of urodynamics on decision-making.

## Methods: participants, interventions and outcomes

### Study setting {9}

We are recruiting 1096 women aged ≥ 18 years, with refractory OAB symptoms, across approximately 60 secondary and tertiary care hospitals in the UK.

### Eligibility criteria {10}

Women aged ≥ 18 years with refractory OAB or urgency predominant MUI (i.e. in whom OAB are their most bothersome symptoms), and

- Have failed conservative management (as per NICE guideline, e.g. pelvic floor muscle training/ bladder retraining) and
- Have failed or have not tolerated pharmacological treatment (at least 2 different drugs) unless contraindicated and
- Are being considered for invasive treatment.

The exclusion criteria are:

- Predominant SUI symptoms;
- Previous urodynamics in the last 12 months;
- Current pelvic malignancy or clinically significant pelvic mass;
- Bladder pain syndrome;
- Neurogenic bladder (e.g. Parkinson’s disease, spinal injuries);
- Urogenital fistulae;
- Previous treatment with BoNT-A/SNM for UI;
- Previous pelvic radiotherapy;
- Prolapse beyond introitus;
- Pregnant or planning pregnancy;
- Recurrent urinary tract infection (UTI) where a significant pathology has not been excluded;
- Inability to give an informed consent.

### Who will take informed consent? {26a}

Members of the local research team trained in good clinical practice will obtain signed consent forms from the study participants in all centres. We will check, sign and date with the date of receipt consent forms that are returned by post. No study-specific activities will take place before consent is given.

### Additional consent provisions for collection and use of participant data and biological specimens {26b}

The patient information leaflet (PIL) refers to further embedded qualitative research within the FUTURE study. The participants will indicate on the study consent form if they accept /or not to be contacted by the qualitative research team for the qualitative study. The qualitative researcher will take verbal consent for the qualitative interviews when the interview is conducted. In addition, the PIL and consent form refer to the possibility of, and seek the participants’ consent to, being contacted for longer-term follow-up of the FUTURE study to further assess the clinical and cost-effectiveness outcomes.

## Interventions

### Explanation for the choice of comparators {6b}

Urodynamics has become standard clinical care without solid evidence to support its routine or selective use, as highlighted by the NICE guideline CG171 [11]; therefore, a superiority type design was adopted to provide the first randomised trial evidence to confirm or refute the clinical and cost-effectiveness of urodynamics over comprehensive clinical assessment only.

### Intervention description {11a}

We are comparing “urodynamics and comprehensive clinical assessment” versus “comprehensive clinical assessment only”. The urodynamics and comprehensive clinical assessment arm refers to the comprehensive invasive and non-invasive assessment of women with urinary symptoms and includes:

- Cystometry
- Free uroflowmetry ± pressure flow studies ± bladder scan.
- Detailed medical history (assessment of urinary symptoms (storage, filling and incontinence symptoms and the most bothersome urinary symptoms), previous investigations and/or treatments (conservative, pharmacological and or surgical) for UI and OAB and past medical or surgical history of relevance)
- Clinical examination including assessment for stress urinary incontinence, pelvic organ prolapse and pelvic masses and other pelvic pathology
- Bladder diary for 3 days to assess daytime frequency, nocturia, urgency and UUI episodes (N.B. A minimum of 24 h completed diary will be accepted as a valid diary. Diary completed at a previous clinic visit within last 3 months will be accepted)

The comprehensive clinical assessment only arm includes a detailed medical history, clinical examination, bladder diary (as outlined above) and post-voiding residual urine volume using ultrasound bladder scanning. Some units may also perform non-invasive free uroflowmetry.

### Treatment pathways following intervention

In the urodynamics plus comprehensive clinical assessment arm the treatment pathway is guided by the urodynamics diagnosis and is in line with the NICE guideline (CG171; See Additional file 1 Treatment Pathway A). The latter recommends BoNT-A 200 units as the first treatment; however since its publication, further evidence confirmed the efficacy of BoNT-A treatment at the lower dose of 100 units with less adverse events [35] and it has since been licensed in that dose. Note: Subsequent NG123 also recommended initial treatment with 100 units [13].

NICE CG171 also recommends offering SNM treatment for patients who are unable or unwilling to perform CISC or following unsuccessful BoNT-A treatment pending MDT discussion [11]. However, in view of the lack of robust evidence on the best sequence of treatments in women with refractory OAB, participants in both arms of the FUTURE study can be offered either BoNT-A (100 units) or the SNM test; the decision will be discussed in the local MDT or as per local standard best practice. This approach will vary between units depending on their local clinical practice and the availability of treatments. Participants with other diagnoses on urodynamics would be offered the appropriate treatments.

Depending on the clinical outcome of treatment, we will offer participants with persistent or de novo symptoms urodynamic tests and treatment accordingly or further/ repeat treatment according to comprehensive clinical assessment only.

In the comprehensive clinical assessment only arm, the treatment pathway is guided by the clinical diagnosis and non-invasive tests (See Additional file 1 Treatment Pathways B). Considering the evidence above, we will offer participants with clinically diagnosed refractory OAB or urgency predominant MUI either BoNT-A (100 units) or SNM test. We will discuss the decision in the local MDT or as per local standard best practice. This approach will vary between units depending on their local clinical practice and the availability of treatments. We will offer participants with other clinical diagnoses (such as overflow incontinence or SUI predominant MUI) and other appropriate treatments such as CISC, SUI surgery or other medical/conservative treatments as per local standard best practice.

Depending on the clinical outcome of initial treatment, we will offer participants with persistent or de novo symptoms urodynamics and treatment accordingly or may consider repeat/further treatment according to comprehensive clinical assessment only (e.g. we may offer patients with persistent OAB symptoms following treatment with 100 units of BoNT-A treatment the option of repeat BoNT-A treatment).

Deviation from the treatment pathways may occur depending on the local clinical practice and the availability of treatments in the participating units. However, the Chief Investigator (CI) or delegate will assess patterns of deviations.

### Intervention data to be collected

We will collect intervention data on case report forms (CRFs). For both arms, this includes data from the detailed medical history, clinical examination and 3-day bladder diary, as well as a clinical diagnosis (OAB versus MUI).

For the urodynamics plus comprehensive clinical assessment arm, we will also collect data from the urodynamics test, including urodynamics diagnosis (DO/MUI/USI/others), voiding assessment on free uroflowmetry (voided volume, post-voiding residual urine volume, voiding pattern and maximum and average flow rate), voiding assessment on pressure flow studies (if performed) and maximum urethral closure pressure on urethral pressure profile (if performed).

For the comprehensive clinical assessment arm, we will collect data on the post-voiding residual urine volume using ultrasound bladder scanning (and/or non-invasive free uroflowmetry if performed).

### Criteria for discontinuing or modifying allocated interventions {11b}

There are no special criteria for discontinuing or modifying allocated interventions. However, in both study arms, we will offer participants evidence-based treatments for refractory OAB according to their diagnosis and as per the defined treatment pathways which were developed in line with the NICE guideline CG171 [11].

### Strategies to improve adherence to interventions {11c}

To be assured of good quality measurements and accurate urodynamics data recording, we have developed a “FUTURE study Guide for Urodynamics Best Practice” (Additional file 2) in conformity with the 2016 International Continence Society (ICS) Good Urodynamics Practices [36].

We have developed clear guidance for urodynamic trace marking to standardise the points used for data in each study and make central reading/audit of traces more reliable.

Prior to performing the first randomised urodynamics test within the FUTURE Study, collaborating units are required to

- Undertake urodynamics machine calibration checks for measurements, and
- Submit two anonymous urodynamics traces with their reports for central reading and review by a panel of experts within the FUTURE study team. Feedback is given to centres for any improvement steps needed.

During the course of the study:

- Collaborating units will submit copies of urodynamics traces/reports for all participants that are randomised to the urodynamics arm for archiving as study data.
- Random central check of traces/reports will be undertaken after ten traces/reports are submitted per unit (five for low recruiting units) by the panel above for quality assurance. If required, one to one feedback will be provided and closer monitoring (random central checks after five traces/reports) undertaken (Additional file 2).

Web-based training on best urodynamics practice is available for collaborating units. An expert clinical engineer (co-investigator) provides one to one support for collaborating units if/when required.

### Relevant concomitant care permitted or prohibited during the trial {11d}

Usual care for participants continues throughout the trial. No relevant concomitant care is prohibited.

### Provisions for post-trial care {30}

Standard care is provided within the UK National Health Service.

### Outcomes {12}

The primary outcome measure is participant-reported success at 15 months post-randomisation (approximately 12 months post-treatment) as measured by the PGI-I.

The PGI-I is a validated single item questionnaire designed to assess the participant’s impression of changes in her urinary symptoms. The PGI-I asks the participant to best describe her urinary symptoms, compared with how they were before the study intervention, on a 7-point scale scored as: (1) “very much improved,” (2) “much improved,” (3) “improved,” (4) “same,” (5) “worse,” (6) “much worse,” or (7) “very much worse. “Success” is defined as responses of “very much improved” or “much improved”; this will capture whether the women are satisfied with their treatment.

The primary economic outcome is the incremental cost per QALY gained of urodynamics and comprehensive clinical assessment compared to comprehensive clinical assessment only, modelled over the lifetime of the patients.

Secondary outcome measures are as follows:

- ○ A less strict definition of success at 15 months derived from the PGI-I where success is defined as a response of “very much improved”, “much improved”, or “improved”.
- ○ Proportion of women receiving invasive treatment at 6 and 15 months post-randomisation.
- ○ Participant-reported outcomes at 3, 6 and 15 months post-randomisation including:
  ▪ OAB symptoms measured by the International Consultation on Incontinence Questionnaire Overactive Bladder (ICIQ-OAB) and the Urgency Perception Scale (UPS);
  ▪ Urgency and UUI episodes measured using the bladder diary;
  ▪ Other urinary symptoms measured using the 3 domains of the International Consultation on Incontinence Questionnaire Female Lower Urinary Tract Symptoms (ICIQ-FLUTS; filling, voiding and incontinence) and the bladder diary;
  ▪ Generic health-related QoL status measured using general (EQ-5D-5L) and condition specific (International Consultation on Incontinence Questionnaire Lower Urinary Tract Symptoms Quality of Life; ICIQ-LUTSqol) QoL assessment tools.
- ○ Adverse events:
  ▪ All serious adverse events;
  ▪ UTI requiring antibiotic treatment;
  ▪ For subsequent treatments:
  - ○ BoNT-A treatment: urinary retention requiring clinical intervention (e.g. catheterisation); CISC.
  - ○ SNM: infection of the SNM lead; lead migration, revision of surgery; wound infection.
  - ○ SUI surgery: Bladder injury; intra operative bleeding requiring return to theatre; post-operative wound infection; nerve injury; tape exposure/extrusion into vagina/lower urinary tract; tape excision/division; tape infection; new onset and related pelvic pain; urinary retention requiring intervention (e.g. catheterisation); CISC; others.
- ○ Qualitative study outcomes:
  ▪ Participants’ attitudes to invasive testing and expected outcomes (prior to randomisation or knowing their allocated study group);
  ▪ Participants’ attitudes to potential treatment options (prior to randomisation or knowing their allocated study group);
  ▪ Participants’ experience of urodynamics and opinions regarding treatment outcome to include evaluation of treatment satisfaction or desire for further treatment (3 to 6 months post-treatment);
  ▪ Surgeon attitudes to the influence of urodynamics on decision-making (at start of the study and 6 to12 months after starting recruitment at their sites).

- ○ Secondary economic outcomes include:
  ▪ Incremental cost per QALY gained of urodynamics and comprehensive clinical assessment compared to comprehensive clinical assessment only up to 15 months;
  ▪ Incremental cost per QALY gained of BoNT-A vs SNM as the initial treatment for refractory OAB over the lifetime of patients;
  ▪ Incremental cost per QALY gained of SNM test and BoNT-A treatment according to clinical assessment only compared to treatment guided by urodynamics over the lifetime of patients;
  ▪ Expected value of perfect information and associated partial values over the lifetime of patients.

### Participant timeline {13}

See Fig. 1 for the participants’ timelines through the trial.

### Sample size {14}

A survey of the collaborating units showed that in clinical practice, the majority of women with refractory OAB are initiated on BoNT-A treatment (60–70%) compared to SNM (15–20%) or other/no treatments (10–25%). In addition, Rovner et al. and Chapple et al. both showed a success rate of around 60% in women with refractory OAB without the urodynamics diagnosis of DO [14, 16]. These two studies defined success differently: Chapple et al. assessed patient-reported success at 12 weeks following injection of 100 units BoNT-A and defined success as greatly improved or improved [16]; Rovner et al. used a dose of 300 units and defined success as no UUI episodes recorded in a 7-day diary recorded at 12 weeks post-treatment [14].

We have also established a consensus amongst clinicians and patient and public involvement groups (PPI) that for urodynamics to be worthwhile, it will need to demonstrate a minimum of 10% superiority over comprehensive clinical assessment only. For 90% power and a 5% level of significance, 986 participants (493/group) are needed using a chi-squared test with continuity correction [37, 38], rising to 1096 (or 548/group) to allow for 10% attrition at 15 months post-randomisation.

### Recruitment {15

Local research teams will identify all potentially eligible women at outpatient clinics or waiting lists for urodynamics/outpatient clinics in each recruiting centre. We may also use participant identification centres (PICs) to identify potential patients. Posters within appropriate clinics will provide information about the study. Local procedures at the participating hospitals will vary and the timing and mode of approach to women and the consent process may accommodate both the specific circumstances at each site and the needs of the women.

We will give or send each eligible woman a PIL describing the FUTURE study and they will have the opportunity to discuss the study with her consultant. Women may also receive their local hospital PIL regarding the urodynamics investigation. Women will have the opportunity to discuss all aspects of the proposed research with the local clinical team (consultant/staff at clinics), the research nurse (RN), and if appropriate, general practitioner, family and friends.

Women may make a decision to participate during an initial consultation with their consultant or during a subsequent visit to hospital (e.g. a clinic appointment) or alternatively at home.

The local RN may telephone eligible women to discuss any queries and arrange a baseline assessment visit. For women who decide to participate, we will send or give them the study documents (consent form and baseline questionnaires) to complete at home. They can either send their completed documents (consent form and baseline questionnaire) through the post to the local team at their treating hospital or bring it with them if they are returning to hospital for another consultation or assessment.

We will keep a log of all potentially eligible patients assessed in order to document the reasons for non-inclusion in the study (e.g. reason they were ineligible or declined to participate) to inform the Consolidated Standards of Reporting Trials (CONSORT) diagram. We will record brief details of potentially eligible patients in the screening logs at each site (these are an aid to monitoring potential participant inclusion). We will assign all women who enter the study a unique study number.

We will send participants who also consent to the qualitative research a dedicated PIL for the qualitative study interviews. This will be followed by an email and/or phone call from a qualitative team researcher to answer any of the participants’ queries.

## Assignment of interventions: allocation

### Sequence generation {16a}

Eligible and consenting participants will be randomised after completing the baseline assessment to either “urodynamics and comprehensive clinical assessment” or “comprehensive clinical assessment only” using the randomisation application at the trial office at the Centre for Healthcare Randomised Trials (CHaRT), University of Aberdeen.

This randomisation application is available 24 h a day, 7 days a week as a web-based application. The randomisation uses stratified random permuted blocks with (a) centre, and (b) diagnosis of OAB versus Mixed Urinary Incontinence used as stratum. In addition, a random component is used in the minimisation algorithm to ensure concealment of the allocation.

### Concealment mechanism {16b}

The web-based randomisation system ensures allocation concealment.

### Implementation {16c}

The Principal Investigator (PI) at site, or member of the local research team (with delegated authority), will access the web-based system. They will enter stratification characteristics into the web-based system, which returns the allocation status. We will inform participants of their allocated pathway following randomisation. If the participants are not present at the time of randomisation, the research team will contact the participant to inform them of the allocated pathway after randomisation. We will inform participants who have consented to take part in the embedded qualitative study of their allocated pathway after their initial qualitative interview.

## Assignment of interventions: Blinding

### Who will be blinded {17a}

Participants, clinical staff or the central trial team cannot be blinded to the allocated procedure because of the nature of the interventions.

### Procedure for unblinding if needed {17b}

There are no requirements for emergency unblinding procedures.

## Data collection and management

### Plans for assessment and collection of outcomes {18a}

We will collect data at baseline and at 3, 6 and 15 months post-randomisation (see Table 1). We will also collect intervention data for each participant.

**Table 1:** Source and timing of outcome measures to be assessed

Outcome measure	Source	Timing			
		Baseline^**a**^	Post-randomisation (months)		
			3	6	15
Treatment success PGI-I	PQ		✓	✓	✓
Generic health status	PQ	✓	✓	✓	✓
EQ-5D-5L					
Condition specific quality of life	PQ	✓		✓	✓
ICIQ-LUTSqol					
Urinary symptoms	PQ	✓	✓	✓	✓
ICIQ-OAB					
ICIQ-FLUTS					
UPS: Urgency Perception Scale					
Urgency and urgency urinary incontinence episodes on (3-day bladder diary)	PQ	✓		✓	✓
Bladder scan	CRF	✓			
Interventions received	CRF, PQ		✓	✓	✓
Adverse events	CRF, PQ		✓	✓	✓
NHS primary and secondary healthcare use	CRF, PQ	✓		✓	✓
Participant resource use	PQ	✓		✓	✓

*CRF* case report form, *PQ* participant-completed questionnaire

^a^Baseline is after informed consent has been given but prior to randomisation

We will collect qualitative data via face to face participants’ interviews where possible, with telephone interviews included for remote study sites, and carried out by an experienced qualitative researcher.

### Plans to promote participant retention and complete follow-up {18b}

We offer and use all methods of delivery and collection of questionnaires and reminders including use of research teams for time points associated with hospitalisation, post, e-mail, web-based and SMS text, taking into account each participant’s stated preferred means of receiving and completing the measures (recorded on the participant contact preference form).

We will send up to three reminders to participants by post, email, phone or text message, taking into account any preferences they may have for mode of communication.

We will send a small token of appreciation (gift voucher(s) of modest value up to £15) to participants on receiving their completed follow-up questionnaires, unless they opt out on the study consent form.

### Data management {19}

The local research team enters locally collected data in the centres. Staff in the trial office will work closely with local research teams to ensure the data are as complete and accurate as possible.

Follow-up questionnaires to participants are sent from and returned to the trial office in Aberdeen. Extensive range and consistency checks are designed to further enhance the quality of the data.

### Confidentiality {27}

Data collected during the course of the research is kept strictly confidential and accessed only by members of the trial team and may be looked at by individuals from the Sponsor organisation or NHS site for the purposes of monitoring and audit.

Participants are allocated a unique study number. Participant’s details are stored on a secure database under the guidelines of the 1998 Data Protection Act. To comply with the 5th Principle of the Data Protection Act 1998, personal data will not be kept for longer than is required for the purpose for which it has been acquired. The CHaRT senior IT manager (in collaboration with the CI) manages access rights to the data set. We anticipate that anonymised trial data may be shared with other researchers to enable international prospective meta-analyses.

### Plans for collection, laboratory evaluation and storage of biological specimens for genetic or molecular analysis in this trial/future use {33}

There are no plans for the collection, laboratory evaluation or storage of biological specimens for genetic or molecular analysis in this trial.

## Statistical methods

### Statistical methods for primary and secondary outcomes {20a}

We will compare primary and secondary outcomes using generalised linear models, with adjustment for the minimisation covariates (centre and diagnosis of OAB versus MUI). We will use sensitivity analyses to explore additional adjustment of healthcare professional effects which arise from a better diagnosis being made due to the use of urodynamics. This is similar to adjusting for surgeon effects when there is more than one surgeon within a centre.

The primary outcome is participant-reported success as measured by the PGI-I at 15 months post-randomisation (approximately 12 months post-treatment). For the primary analysis, the PGI-I responses will be dichotomised to “success” defined as “very much improved” or “much improved” as this is felt to be the best categorisation of whether the women are satisfied with their treatments and interventions. We will use a repeated measures mixed effects logistic regression, including the 6-month measurement to increase the power to estimate the treatment effect at 15 months post-randomisation.

We will analyse secondary outcomes using the appropriate linear model. For example, we will analyse the less strict definition of success using a repeated measures mixed effects logistic regression in the same way as the primary outcome will be analysed. We will also analyse the proportion of women receiving invasive treatment using a logistic regression. We will analyse continuous outcomes such as the ICIQ-FLUTS scores and quality of life scores and the EQ-5D-5L using a repeated measures mixed effects linear regression.

We will make available the statistical analysis plan and the health economy analysis plan as a separate publication(s).

### Quality-adjusted life years

We will estimate QALYs using the EQ-5D-5L tariff that is recommended by NICE at the time of the analysis; this is currently the van Hout “cross-walk” tariff [39]. We will estimate QALYs using linear interpolation between time points. We will undertake exploratory analysis to assess the QALY loss related to urodynamics (e.g. anxiety and discomfort) by estimating the degree to which EQ-5D-5L values at 3 months post-randomisation are affected by time since urodynamic testing. If a robust estimate of QALY loss is produced, we will examine the impact of its incorporation into the cost-effectiveness analysis using a sensitivity analysis.

### Within trial cost-effectiveness analysis

The within trial analysis will follow the best practice guidelines [40]. The analysis will calculate total costs and QALYs for each patient and estimate the incremental costs and QALYs using a seemingly unrelated regression model with baseline covariates including age and baseline EQ-5D-5L score and missing data imputed using multiple imputation [41, 42].

We will base the cost-effectiveness acceptability curves on the adjusted and imputed analysis described above. We will undertake deterministic sensitivity analyses to look at three sources of methodological uncertainty; societal perspective, the EQ-5D-5L tariff and any QALY loss associated with urodynamics identified in the exploratory analysis described above. Undertaking a sensitivity analysis with the EQ-5D-5L tariff is required as alternative tariffs are available; the current recommended tariff is the van Hout “cross-walk” tariff [43]

### Patient lifetime cost-effectiveness analysis

The primary analysis is model based. Such an approach is considered superior to trial-based analyses as it can be designed to better fit the research question and incorporate other relevant sources of data [44]. In this particular situation, the model can incorporate the longer-term costs and consequence of using urodynamics which cannot be observed in the trial. However, the first 15 months of the model will be based on the trial results. The structure of the model beyond 15 months will be based on a pre-existing model [18]. A key improvement over this model is the use of more appropriate utilities taken from the trial. In addition, we will undertake targeted literature searches to assess whether any relevant new studies have been published for the other parameters.

In line with the Rachaneni model [18], exploratory analyses examine whether targeted urodynamics for a subgroup of patients has the potential to be cost-effective. In addition to Rachaneni, we will undertake two exploratory analyses:

- A non-randomised comparison of the cost-effectiveness of different sequence of treatments (i.e. initiated on BoNT-A vs SNM).
- A non-randomised comparison of the cost-effectiveness of SNM according to clinical assessment only compared to treatment guided by urodynamics.

These two comparisons take account of the lack of randomisation using methods consistent with those of the analogous clinical analyses.

We will also undertake a probabilistic sensitivity analysis on the modelled results and its associated incremental cost-effectiveness ratio, cost-effectiveness plane and cost-effectiveness acceptability curves generated. We will undertake a value of information analysis using SAVI (http://savi.shef.ac.uk/SAVI/). The partial values are used to identify those parameters where there is greatest value in resolving outstanding uncertainty. We will undertake a deterministic sensitivity analyses to look at the three sources of methodological uncertainty; societal perspective, the EQ-5D-5L tariff and the length of disutility associated with urodynamics.

### Qualitative interviews

A standardised approach will be employed in accordance with published qualitative research methods [45–49].

Interviews are semi-structured and follow a topic guide informed by literature review and discussion between study researchers and encourage participants to discuss their perspectives with regard to the qualitative study aims. Interviews are audio recorded, transcribed verbatim (including descriptions of non-verbal factors where appropriate) and uploaded onto a qualitative software package (QSR Nvivo 10) to aid data management. The qualitative researcher will conduct the analyses according to principles of thematic content analysis.

We will use theoretical purposive (non-probability) sampling to ensure the diverse characteristics of the population are sampled (e.g. participants varying in age, relevant clinical history (MUI vs OAB), investigations received (urodynamics and comprehensive clinical assessment versus comprehensive clinical assessment only), and treatments received (SNM vs. BoNT to include day case vs. local anaesthetic procedures). Sampling and analyses continue in iterative cycles until data saturation is achieved. It is anticipated a minimum of thirty to forty patient interviews will be undertaken to effectively capture the opinions of those in both arms of randomisation, the numerous potential treatments and treatment considered successful and failed. Approximately ten to fifteen clinician interviews are proposed to explore the clinical aspects of urodynamics with regard to clinical decision-making.

### Interim analyses {21b}

There will be no interim analyses.

### Methods for additional analyses (e.g. subgroup analyses) {20b}

We will perform a secondary supporting analysis of the primary outcome using ordinal logistic regression on the 7-point PGI-I scale. This will use a generalised ordered logit model with the partial proportional odds model (for example, as implemented in “gologit2” in Stata) to relax this restrictive assumption of the full proportional odds model. We will also include a suitably defined per protocol analysis as a secondary supporting analysis.

We will compare the outcomes in pre-specified subgroups of participants with OAB vs MUI. In addition, we will explore, in a non-randomised analysis, the following:

1. Clinical and cost-effectiveness of the different treatment pathways of those initiated on BoNT-A treatment vs those initiated on SNM treatment.
2. Clinical and cost-effectiveness of SNM treatment according to clinical assessment only compared to treatment guided by urodynamics.
3. Clinical and cost-effectiveness of BoNT-A treatment according to clinical assessment only compared to treatment guided by urodynamics

### Methods in analysis to handle protocol non-adherence and any statistical methods to handle missing data {20c}

Analysis will be by intention-to-treat. We will assess how robust all findings are to any missing data (anticipated to be no more than 10% for the primary outcome) using multiple imputation approaches under an assumption of missing at random. We will consider non-ignorable missing data mechanisms if the patterns of missing data across the two randomised groups suggest this is appropriate.

### Plans to give access to the full protocol, participant-level data and statistical code {31c}

The full protocol is available on the funder’s website (https://www.journalslibrary.nihr.ac.uk/programmes/hta/1515005/#/). Non-identifiable participant-level data may be available on request to the Chief Investigator (CI), Professor Mohamed Abdel-fattah (m.abdelfattah@abdn.ac.uk)

## Oversight and monitoring

### Composition of the coordinating centre and trial steering committee {5d}

The Trial Office is in CHaRT based within the Health Services Research Unit, University of Aberdeen and provides day to day support for the clinical centres. The Trial Manager takes responsibility for the day to day transaction of trial activities, for example approvals, site set-up and training, oversight of recruitment and follow-up rates. The Data Coordinator provides clerical support to the trial, including organising all aspects of the questionnaires (mailing, tracking and entering returned data using the trial web data entry portal).

The FUTURE Trial Office Team (CI, trial manager, data coordinator, statistician) meets formally, approximately fortnightly during the course of the study to ensure smooth running and trouble-shooting, but more frequently during the set-up phase as required.

The trial is supervised by its Project Management Group (PMG). The group consists of grant holders (clinicians, statisticians, health economists and qualitative researchers) and representatives from the Trial Office. Observers are invited to attend at the discretion of the PMG. The PMG meets approximately every 2 months in the first and last 6 months of the trial and approximately every 3 months in-between.

The PMG has the expertise to cover the clinical, methodological and management aspects of the FUTURE study.

Any modification to the project is normally discussed by the PMG, and when relevant by the Trial Steering Committee (TSC) and is approved by the Sponsors and funder before application to the Research Ethics Committee (REC) and Research and Development (R&D). An exception to the above is in the case where an immediate implementation of safety measures is required; the Sponsor is then notified as soon as possible.

A TSC oversees the conduct and progress of the FUTURE study. The TSC meets annually and includes an independent chair, clinical and methodological expertise and a lay representative.

### Composition of the data monitoring committee, its role and reporting structure {21a}

An independent Data Monitoring Committee (DMC) oversees the safety of participants in the FUTURE study. The DMC Charter documents the terms of reference of the DMC and the names and contact details. This is filed in the Trial Master File. The committee meets every 6 to 12 months to monitor the study data and make recommendations as to any modifications that are required to be made to the protocol or the termination of all or part of the study. The study has adopted the DAMOCLES Charter for DMCs [50].

### Adverse event reporting and harms {22}

In FUTURE, we will only record adverse events (AEs) and serious adverse events (SAEs) related to the study interventions. A serious adverse event is defined as any AE that results in death, is life threatening (i.e. the subject was at risk of death at the time of the event; it does not refer to an event which hypothetically might have caused death if it were more severe), results in persistent or significant disability or incapacity, requires hospitalisation or prolongation of existing hospitalisation or is otherwise considered medically significant by the investigator.

All AEs and SAEs meeting the criteria for recording within the trial are recorded from the time a participant consents to join the trial until the end of their follow-up period. Every follow-up visit and questionnaire will inquire on expected AEs/SAEs. In addition, we will use open ended and non-leading verbal questioning of the participant to enquire about AE/SAE occurrence or re-admission to hospital and any further treatment received.

Depending on severity, when an AE/SAE meeting the criteria for recording within the FUTURE Study occurs, it is the responsibility of the local PI (or delegate) to review appropriate documentation (e.g. hospital notes, laboratory and diagnostic reports) related to the event. The PI or delegate should then record all relevant information in the CRF (and on the SAE form if required).

PI or delegates are responsible for notifying the trial office of any SAEs that require to be recorded in line with the FUTURE study protocol. If an SAE is recorded on a participant questionnaire, the Trial office liaises with the relevant study site to obtain further information.

When an SAE form is uploaded onto the trial website, the Trial Manager is automatically notified. If, in the opinion of the local PI and/or the CI, the event is confirmed as being *serious* and *related* and *unexpected*, the CI or Trial Manager notifies the sponsor within 24 h of receiving the signed SAE notification. The sponsor provides an assessment of the SAE. A Sponsor cannot downgrade an assessment from the PI or CI. Any disparity is resolved by further discussion between these parties.

The CI or delegate reports any related and unexpected SAEs to the REC within 15 days of the CI becoming aware of it.

### Frequency and plans for auditing trial conduct {23}

The CI/ PMG ensures, through the TSC and Sponsor, that adequate systems are in place for monitoring the quality of the trial (compliance with appropriate governance) and appropriate expedited and routine reports, to a level appropriate to the risk assessment of the trial. CHaRT’s Standard Operating Procedures are followed.

The approach to, and extent of, monitoring are specified in a trial monitoring plan and is appropriate to the risk assessment of the trial. PIs and their host Trusts are required to permit trial related monitoring and audits to take place by the Sponsor and/ or regulatory representatives providing direct access to source data and documents as requested.

### Plans for communicating important protocol amendments to relevant parties (e.g. trial participants, ethical committees) {25}

Any amendments to the project are approved by the Sponsors and funder before application to REC and R&D unless in the case of immediate safety measures being required/ implemented when the Sponsor is notified as soon as possible. Any deviations from the Protocol will be fully documented.

## Dissemination plans {31a}

We will develop a publication and dissemination plan to include conference presentation(s) and journal publication(s).

The findings of the trial will be disseminated nationally through the UK Continence Society (UKCS), British Society for Urogynaecology (BSUG) and British Association of Urological Surgeons (BAUS) and internationally though the European Association of Urology (EAU) and ICS.

BAUS, UKCS and BSUG are the UK specialist bodies with the responsibility of guiding clinical practice, policies, research priorities, governance and training in matters related to UI in women. UKCS provide urodynamics guidance and unit accreditation in the UK. They are well placed to implement the findings by influencing NHS policy and dissemination of evidence-based clinical practice to its members. The results of the trial will be included in updates of NICE and EAU guidelines, which directly influence practice in the UK and beyond.

Our dissemination plans include a Health Technology Assessment (HTA) monograph; presentations at the UKCS, BSUG, BAUS, EAU, ICS annual scientific meetings; publications in high-impact open access peer-reviewed journals; presentations at health economic and health services research meetings; and development of plain English language summaries of our main findings for patient organisations and communities.

We plan to use our PPI partners, Bladder Health UK (Charity Reg No: 1149973A), to ensure this information meets the needs of users, and we shall share our findings with all FUTURE participants. In addition, summaries will be published in health-related media such as Reuters Medical, Uro-today and Nature. We will use social media such as Twitter and Facebook to inform the OAB user groups with a summary of the study results.

## Discussion

The FUTURE trial is a large, pragmatic, multi-centre, superiority randomised controlled trial to determine the effectiveness and cost-effectiveness of routine urodynamics plus comprehensive clinical assessment versus comprehensive clinical assessment only in the management of women with refractory OAB symptoms.

The trial team established collaboration with the largest relevant patient support groups in the UK to provide insights from the patient’s perspective. The Bowel and Bladder Foundation advised on the treatment pathways, proposed assessment tools and outcome measures at time of study design. Unfortunately, they later ceased to exist as an independent PPI group. Bladder Health UK is a grant holder and part of the PMG providing clear leadership on the patient perspective and is integral to the development of the study protocol and all the study documents including the patient information sheet; letters of invitation/reminders; participants’ questionnaires and the bladder diary.

To maximise recruitment into the trial, FUTURE adopted a hub structure. Each hub has approximately 10 allocated centres and is led by a hub leader and a funded part time hub coordinator. The hub leader and coordinator support the trial manager with the set-up of centres, ensure continuous engagement of PIs and research nurses and promote recruitment within their hubs.

As with most clinical trials, recruitment and participant retention are key challenges. The study team has adopted several strategies to promote recruitment and retention within FUTURE. These include the following: the inclusion of Participant Identification centres (PICs), encouragement of collaborating centres to engage both Gynaecology and Urology departments, and organisation of regional hub meetings at which the CI and trial manager attend. We have made a recruitment illustration video which is available on the study website to aid sites through recruitment. We also utilise regular site and participant newsletters to sustain positive engagement with centres and participants. Finally, we opened more centres than originally planned (65 vs planned 38 centres)

NICE CG 171 was the relevant guideline at the time of the FUTURE study planning in 2017. In 2019, NICE updated their guideline on the management of UI in women with the publication of NG123. NG123 slightly amended their recommendation to “consider offering BoNT-A treatment, following MDT discussion, to women with refractory OAB in whom DO was not demonstrated on urodynamics” [13]. There were no changes to the recommendation of routine urodynamics investigations in women with refractory OAB. These changes did not require any change to the FUTURE study protocol.

The results of the FUTURE study are expected to answer the important research question on whether routine urodynamics investigation in addition to comprehensive clinical assessment improve patient-reported outcomes following treatment and is cost-effective, compared to comprehensive clinical assessment only, in women with refractory OAB symptoms. The study results will have an impact on all the major stakeholders involved in the management of refractory OAB in women. The findings are expected to be incorporated into clinical practice guidelines and treatment recommendations from NICE, UKCS, BSUG and EAU. For patients and clinicians, the study results are expected to facilitate and guide treatment decision-making. The results will also provide cost-effectiveness evidence for the NHS, which will facilitate decision-making regarding the allocation of resources for treatment, and provision of services.

## Trial status

The first participant was recruited in November 2017. The current protocol version is 10.0, 01/07/2021. Recruitment was due to complete on 31st May 2020. However, on instruction by the Trial Sponsor, recruitment was suspended on 16th March 2020 due to the COVID-19 outbreak. At this point, 1022 participants had been randomised. Recruitment restarted in August 2020 and the recruitment end date extended to 31st January 2021. Please see additional file 3 for further implications of the COVID-19 pandemic.

## Abbreviations

AE: Adverse event
BoNT-A: Botulinum toxin injection
CHaRT: Centre for Healthcare Randomised Trials
CI: Chief Investigator
CISC: Clean intermittent self-catheterisation
CRF: Case Report Form
DMC: Data Monitoring Committee
DO: Detrusor overactivity
DOI: Detrusor overactivity incontinence
EQ-5D-5L: EuroQol Group’s 5 dimension health status questionnaire
HTA: Health Technology Assessment
ICIQ-FLUTS: International Consultation on Incontinence Questionnaire - Female Lower Urinary Tract Symptoms
ICIQ-LUTSqol: International Consultation on Incontinence Questionnaire – Lower urinary tract symptoms Quality of Life
ICIQ-OAB: International Consultation on Incontinence Questionnaire – Overactive Bladder
ICS: International Continence Society
ISRCTN: International Standard Randomised Controlled Trial Number
MDT: Multidisciplinary team
MUI: Mixed urinary incontinence
NHS: National Health Service
NICE: National Institute for Health and Care Excellence
OAB: Overactive bladder
PGI-I: Patient Global Impression of Improvement
PI: Principal Investigator
PIC: Participant Identification Centre
PIL: Patient Information Leaflet
PMG: Project Management Group
PPI: Patient and Public Involvement
QALY: Quality-adjusted life year
QoL: Quality of Life
R&D: Research and Development
RCT: Randomised controlled trial
REC: Research Ethics Committee
RN: Research nurse
SAE: Serious adverse event
SNM: Sacral neuro-modulation
TSC: Trial Steering Committee
UI: Urinary incontinence
UPS: Urgency Perception Scale
USI: Urodynamic stress incontinence
UTI: Urinary tract infection
UUI: Urgency urinary incontinence
